# Effects of in vitro metabolism of a broccoli leachate, glucosinolates and *S*-methylcysteine sulphoxide on the human faecal microbiome

**DOI:** 10.1007/s00394-020-02405-y

**Published:** 2020-10-16

**Authors:** Lee Kellingray, Gwénaëlle Le Gall, Joanne F. Doleman, Arjan Narbad, Richard F. Mithen

**Affiliations:** 1 Food Innovation and Health, Quadram Institute Bioscience, Norwich Research Park, Norwich, NR4 7UQ UK; 2 Analytical Sciences Unit, Quadram Institute Bioscience, Norwich Research Park, Norwich, NR4 7UQ UK; 3 Gut Microbes and Health, Quadram Institute Bioscience, Norwich Research Park, Norwich, NR4 7UQ UK

**Keywords:** Broccoli, Glucosinolates, *S*-methylcysteine sulphoxide, Human gut microbiome, Lactobacilli, Short chain fatty acids

## Abstract

**Purpose:**

*Brassica* are an important food source worldwide and are characterised by the presence of compounds called glucosinolates. Studies indicate that the glucosinolate derived bioactive metabolite sulphoraphane can elicit chemoprotective benefits on human cells. Glucosinolates can be metabolised in vivo by members of the human gut microbiome, although the prevalence of this activity is unclear. *Brassica* and *Allium* plants also contain *S*-methylcysteine sulphoxide (SMCSO), that may provide additional health benefits but its metabolism by gut bacteria is not fully understood.

**Methods:**

We examined the effects of a broccoli leachate (BL) on the composition and function of human faecal microbiomes of five different participants under in vitro conditions. Bacterial isolates from these communities were then tested for their ability to metabolise glucosinolates and SMCSO.

**Results:**

Microbial communities cultured in vitro in BL media were observed to have enhanced growth of lactic acid bacteria, such as lactobacilli, with a corresponding increase in the levels of lactate and short-chain fatty acids. Members of *Escherichia* isolated from these faecal communities were found to bioconvert glucosinolates and SMCSO to their reduced analogues.

**Conclusion:**

This study uses a broccoli leachate to investigate the bacterial-mediated bioconversion of glucosinolates and SMCSO, which may lead to further products with additional health benefits to the host. We believe that this is the first study that shows the reduction of the dietary compound *S*-methylcysteine sulphoxide by bacteria isolated from human faeces.

**Electronic supplementary material:**

The online version of this article (10.1007/s00394-020-02405-y) contains supplementary material, which is available to authorized users.

## Introduction

The structure of the human gut microbiome is influenced by multiple factors, such as mode of birth [1], age [2], host genotype [3], antibiotic treatment [4], environmental factors [5] and diet [6, 7]. As such, a large variation in the gut microbiome has been observed between individuals [8, 9] and diet is considered to be one of the most influential environmental factors determining the composition of the human gut microbiome [10]. Studies indicate that a long-term diet rich in fibre correlates with an increased abundance of *Prevotella*, whilst a long-term diet containing high levels of protein and fat is associated with higher levels of *Bacteroides* [7, 10]. Short-term dietary interventions can result in rapid changes to the composition of the gut microbiome, but the community tends to revert to the original composition within days after the intervention [6, 10]. This illustrates the elasticity and the range of functional capabilities within the microbiome; as the nutrient profile of the intestinal environment changes, the composition of the bacterial community alters to maximise nutrient utilisation. Lactic acid bacteria (LAB) form part of the gut microbiome and are considered to be beneficial to the host. Although these bacteria have limited biosynthetic capabilities, they are efficient metabolisers of carbohydrates [11]. The major fermentation product is lactic acid, which can be further metabolised to produce other SCFAs [12]. Although SCFAs are bacterial waste products, these compounds have been shown to have beneficial properties for human health [13]. Many Firmicutes, including LAB, have evolved to be tolerant of the acidic microenvironments that are generated at the sites of colonic substrate fermentation [14]. Acid tolerance can give these bacteria a competitive advantage over others, such as *Bacteroides* and strains of *Escherichia coli*, whose growth has been shown to be inhibited at a physiologically relevant pH of 5.5 [14].

Links between the diet, gut microbiome and health-promoting effects have led to an increased interest in identification of functional foods that promote the growth of beneficial bacteria. *Brassica* vegetables, such as broccoli, are considered nutritious and are a rich source of fibres, vitamins, minerals, carotenoids and phenolic compounds [15]. These plants are characterised by the presence of phytochemicals called glucosinolates, which have a role in plant defence through hydrolysis by the plant myrosinase enzyme. However, cooking these vegetables can inactivate the plant myrosinase. Therefore, we rely on the myrosinase-like activity of the gut microbiome to hydrolyse glucosinolates and form bioactive breakdown products, such as isothiocyanates (ITCs). Research has shown that single strains of human gut bacteria from a range of phyla have the ability to hydrolyse glucosinolates [16–19], that bacterial glucosinolate metabolism occurs in vivo with a potential role for enterohepatic recycling [20, 21] and that there are inter-individual differences in glucosinolate metabolism, as evidenced by variation in urinary ITC excretion [22]. Studies have determined that human gut bacteria are able to convert glucosinolates, such as glucoraphanin and glucoiberin, via the reduction of a sulphoxide group [23–25]. It has been postulated that the reduction of glucosinolates may be a mechanism employed by some bacteria to overcome steric hindrance, caused by the presence of the sulphoxide groups rendering the β-thioglucosidic bonds inaccessible to bacterial myrosinase-like enzymes [23]. It was shown that a strain of *E. coli* did not exhibit the ability to hydrolyse glucosinolates until after it had converted them to their reduced analogues [23]. This indicates that there may be multiple mechanisms by which the human gut microbiome can hydrolyse glucosinolates. *S*-methylcysteine sulphoxide (SMCSO) is found in *Brassica* and *Allium* and is thought to function as a phytoalexin, providing protection against microbial pathogens and herbivores through its degradation by cysteine sulphoxide lyases [26, 27]. Bacterial cysteine β-lyase activity has been detected within the human gut microbiome and has been attributed to a diverse range of bacteria, including *E. coli* [28, 29]. The bacterial cysteine lyase has broad substrate specificity and is able to cleave the C–S bonds of a range of *S*-alkyl-cysteine molecules, in a manner similar to the plant cysteine lyases [26]. Research into the beneficial effects of SMCSO and its metabolic products have indicated that this dietary compound may exhibit protective effects against cancer [30–32], diabetes [33] and cardiovascular disease [34, 35].

In the current study, we examined the effects of a broccoli leachate on the composition and function of five human faecal microbiomes and determined SMCSO metabolism by pure culture of bacteria.

## Materials and methods

### Bacterial media

#### Generating a broccoli leachate

Beneforté^®^ broccoli (Marks & Spencers, UK) was steamed in a pre-heated domestic steamer for 3 min, snap-frozen and stored at – 20 °C. The frozen broccoli was lyophilised using a benchtop modulyo freeze drier (Edwards Vacuum) and milled using a domestic coffee-bean grinder. Powdered broccoli was added to 0.22 μm filtered water (50 mg/ml), vortexed and incubated at room temperature for 60 min, prior to centrifugation at 5292 ×*g*, 21 °C, for 10 min. The supernatant was passed through a Whatman GF/D filter under vacuum and the water content was reduced approximately tenfold using a BUCHI Rotavapor R-210 rotary evaporator set to 60 °C, 72 mbar. The concentrated broccoli leachate was filtered under vacuum through a succession of decreasing pore size filters in the following order: Whatman GF/A, Whatman GF/B, nitrocellulose membrane 60 μm, 40 μm and 0.22 μm. A 600 μl aliquot was removed for high-performance liquid chromatography (HPLC) analysis, with the remaining leachate filtered through a Sartolab BT500 0.2 μm bottle top filter unit into a sterile bottle and stored at 4 °C.

#### Preparation of broccoli leachate-containing media and glucose media

Three technical replicates of the broccoli leachate stock solution were analysed using HPLC to identify the concentration of glucoraphanin. Sterility was confirmed by streaking the broccoli leachate on brain heart infusion (BHI) agar plates, which were incubated anaerobically or aerobically at 37 °C. Broccoli leachate was added to Chemostat nutrient media (CNM) (peptone water 2 g/l, yeast extract 2 g/l, NaCl 0.1 g/l, K_2_HPO_4_ 0.04 g/l, KH_2_PO_4_ 0.04 g/l, MgSO_4_·7H_2_O 0.01 g/l, CaCl_2_·2H_2_O 0.01 g/l, NaHCO_3_ 2 g/l, cysteine.HCl 0.5 g/l, bile salts 0.5 g/l, Tween 80 2 ml/l, hemin 0.02 g/l and vitamin K_1_ (0.5 v/v in ethanol) 10 μl/l) to make 30 μmole glucoraphanin broccoli leachate-containing (BL) media (relative to ~ 47.3 g wet weight broccoli) and the 30 μmole glucose media were generated by adding 113.79 mg glucose to 1 l CNM. The bottles of prepared media (47.5 ml) were transferred to an anaerobic cabinet to deoxygenate for 12 h.

### Faecal donors

Faecal material was obtained from five participants recruited onto the ENGAGE study (ClinicalTrials.gov: NCT01927666) who gave written informed consent for their stools to be used in these experiments and the resultant data to be published in an anonymised form. Details of the faecal donors are displayed in Table 1. The ENGAGE study protocol was approved by the Human Research Governance Committee at Quadram Institute Bioscience (formally the Institute of Food Research) and Hertfordshire Research Ethics Committee (12/EE/0483) and the work described was performed in accordance with the Helsinki Declaration of 1975. All study participants produced a urine sample for urinalysis which was screened for protein, blood, leukocytes, nitrites, glucose, ketones, bilirubin and urobilinogen via a dipstick urine test (Multistix^®^ SG; Siemens). The exclusion criteria included a medical history of gastrointestinal disorders/surgery, long-term medical conditions (e.g. diabetes) or those taking medication, such as laxatives, that would have affected the study outcome, recent (≤ 1 month) or long-term antibiotic use, regular use of over-the-counter medications for gastrointestinal-associated conditions, intermittent prebiotic &/or probiotic usage, the usage of dietary supplements or herbal remedies that would have affected the study outcome and regular/recent use of colonic irrigation or other bowel cleansing techniques.

**Table 1:** Age, gender, body mass index (BMI) and smoking status of the donors

Faecal donors	Age (years)	Gender	BMI (kg/m^2^)	Smoker
Donor 1	44	Male	24.1	N
Donor 2	24	Female	23.3	N
Donor 3	52	Female	20.9	N
Donor 4	50	Female	38.4	N
Donor 5	61	Female	20.5	N

### Culturing human faecal bacteria

Fresh faeces (17 g), voided ~ 2–4 h previously, was placed into a stomacher strainer bag (Seward) and deoxygenated PBS was added to obtain a mass of 170 g. The faecal matter was homogenised using the Stomacher 400 circulator (Seward, UK) set to 230 rpm, for 45 s. A 100 ml aliquot of the homogenised faecal slurry was transferred into an anaerobic cabinet set at 37 °C, mixed and 8 ml aliquots were stored for 16S rRNA gene analysis. Aliquots (2.5 ml) of the faecal slurry were added to the four replicates of each deoxygenated media (47.5 ml). The lids of the glass bottles were slightly loosened to allow for gas exchange and following a 12 h anaerobic static incubation, aliquots were taken for analyses. A 2.5 ml aliquot was transferred from each replicate to freshly prepared media (47.5 ml) to start the next cycle of culturing. Three 2 ml aliquots, collected from each replicate after each 12 h cycle, were centrifuged at 9600×*g* for 5 min and the supernatants were filter-sterilised and stored at − 80 °C for HPLC, LC–MS/MS and ^1^H NMR spectroscopy analysis. An 8 ml aliquot, collected from each replicate after each 12 h cycle, was centrifuged at 23,500×*g*, at 4 °C, for 15 min and the pellets were stored at − 80 °C for 16S rRNA gene metataxonomic analysis. The sample collection and spiking of fresh media was repeated as described above for four 12 h cycles. The metataxonomic and ^1^H NMR results from donor 1 indicated that pH measurements may assist data interpretation, therefore the pH of the cultured microbiomes were recorded for donors 2–5. At the end of cycle 1 and 4, pH measurements of the cultured microbiomes for donors 2–5 were recorded using a Hanna HI98103 pH meter (Hanna Instruments, UK). This experimental design was used to culture five independent human faecal samples.

### Metataxonomic analysis

The DNA of the cultured microbiome was extracted using the FastDNA SPIN Kit for Soil (MP Biomedicals, UK) with a bead-beating step [36]. DNA quality was assessed using 1% agarose gel electrophoresis and quantified with a NanoDrop ND-1000 UV/vis spectrophotometer (NanoDrop Technologies, Inc., USA). The samples were sent to the Animal Health and Veterinary Laboratories Agency (UK), where the V4 and V5 regions of the 16S rRNA genes were amplified using the U515F (5′-GTGYCAGCMGCCGCGGTA) and U927R (5′-CCCGYCAATTCMTTTRAGT) primers. The amplicons were sequenced using 454 pyrosequencing, as described by Ellis et al. [37]. Sequencing reads were analysed using the Quantitative Insights Into Microbial Ecology (QIIME) 1.8 software and RDP classifier (version 2.10) 16S rRNA gene sequence database [38, 39]. All sequences were filtered to meet the following criteria: read length within 200 and 1000 bp; a maximum of 6 ambiguous bases; a minimum average quality score of 25 within a 50 bp window; and exact match to primer sequences. The trimmed reads were filtered for chimeric sequences using ChimeraSlayer, bacterial taxonomy assignment with a confidence value threshold of 50% was performed with the RDP classifier and trimmed reads clustered into operational taxonomic units at 97% identity level.

### ^1^H nuclear magnetic resonance spectrometry

Samples (2 ml) were centrifuged at 9600×*g* at room temperature for 5 min. The supernatants were passed through a 0.2 μm syringe filter and stored at − 20 °C. The samples were thawed at room temperature, mixed and 600 μl was added to 70 μl of 0.4 mM phosphate buffer (K_2_HPO_4_ and NaH_2_PO_4_ [pH 7.4]) made up in 100% D_2_O, containing 0.1% NaN_3_ (104 mg) and 2.5 mM sodium 3-(Trimethylsilyl)-propionate-*d*_4_, (TSP) 44.5 mg as a chemical shift reference. The samples were mixed and 600 μl was transferred into a 5 mm NMR tube for spectral acquisition as previously described [40]. Each ^1^H NMR spectrum was acquired with 64 scans, a spectral width of 12,500 Hz, an acquisition time of 2.62 s and a relaxation delay of 3 s.

### Glucosinolate analysis

Glucosinolates were measured using an adapted method that converts glucosinolates to their corresponding desulphoglucosinolates [41]. Briefly, 200 μl of the samples were added to 4.8 ml of 70% MeOH (70 °C) and 50 μl of 16 mM sinigrin was added to act as an internal standard. Aliquots (3 ml) were added to ion exchange columns, washed with 0.22 μm filtered water and incubated overnight following the addition of purified sulphatase (75 μl). The desulphoglucosinolates were eluted with 0.22 μm filtered water and analysed using HPLC as described by Saha et al. [25].

### Glucosinolate hydrolysis product analysis

Glucosinolate hydrolysis products and their conjugates were analysed using a Phenomenex Luna C18(2) (100 × 2 mm id, 3 μm particle size) column connected to a model 1290 infinity 6490 Triple Quad LC–MS/MS system (Agilent Technologies) comprised of a degasser, binary pump, cooled autosampler, column oven, diode array detector and 6490 mass spectrometer. The samples were eluted at 0.25 ml/min with a gradient of increasing acidified ACN: solvent A (0.1% ammonium acetate buffer), solvent B (ACN with 0.1% acetic acid). The gradient increased from 5% acidified ACN to 100% over 9.1 min, prior to re-equilibration to 5% acidified ACN for 2.9 min. The LC eluent flow was sprayed into the mass spectrometer interface without splitting. ITCs and their conjugates were monitored by tandem MS using multiple reaction monitoring (MRM) with electrospray ionisation source in the positive ion mode. Identification was achieved on the basis of retention time and product ions and quantification was performed through the use of calibration standards.

### Bacterial isolation from cultured microbiome

Following the fourth 12 h cycle, the cultures of each media were streaked onto BHI agar plates and incubated under anaerobic conditions at 37 °C for 48 h. Bacterial isolates were transferred to a single well of a 96-well microtitre plate containing 300 μl of 30 μmole BL and GL media, respectively, and incubated overnight under anaerobic conditions at 37 °C. The contents of each well were transferred to sterile tubes containing 500 μl of 50% glycerol and stored at − 80 °C. The isolates were revived by spotting 2 μl onto MRS agar plates in duplicate, with each incubated in an anaerobic cabinet or a static incubator, both at 37 °C for 12 h.

### Bacterial isolate identification

Colony polymerase chain reaction (PCR) was performed on 10 colonies. Each colony was transferred to a 50 μl PCR tube containing 10 μl of 0.22 μm filtered water and resuspended. The resuspended colonies were boiled in a pre-heated Hybaid PCR Sprint thermal cycler (Thermo Scientific) set at 95 °C, for 5 min and used as template DNA. The PCR reaction mixture was generated by adding the following together: 10 μl 5 × colourless GoTaq reaction buffer (Promega), 0.4 μl (25 mM) dNTP mix (Thermo Scientific), 1 μl (20 μM) universal forward primer (AmpF 5′- GAGAGTTTGATYCTGGCTCAG -3′) [42], 1 μl (20 μM) universal reverse primer (AmpR 5′- AAGGAGGTGATCCARCCGCA -3′) [42], 36.35 μl 0.22 μm filtered water, 1 μl template DNA and 0.25 μl GoTaq DNA polymerase (Promega), to make a final volume of 50 μl per reaction mixture. A negative control was generated as above with the omission of template DNA and increasing the volume of 0.22 μm filtered water to 37.35 μl. The thermal cycler was programmed to perform the amplifications as previously described by Nueno-Palop & Narbad [43] with minor modifications: one cycle of 95 °C for 2 min followed by 25 cycles of 95 °C for 30 s, 55 °C for 30 s, 72 °C for 60 s and one cycle of 72 °C for 5 min. The PCR products were resolved by electrophoresis in a 1% (w/v) agarose gel and visualised by ethidium bromide staining. PCR amplified products were cleaned using QIAquick PCR Purification Kit (Qiagen) according to the manufacturer’s instructions.

Two 15 μl aliquots of each cleaned PCR amplicon was added to a Eurofins Mix2Seq tube for 16S rRNA gene sequencing, with 2 μl of the AmpF primer added to one aliquot and 2 μl of the AmpR primer added to the other aliquot. The paired samples were assembled to form a single contig using SeqMan (DNASTAR, Inc.) and checked for errors or mismatches using FinchTV (Geospiza, Inc.). The quality-checked aligned sequences were uploaded to the online RDP SeqMatch tool (https://rdp.cme.msu.edu/seqmatch/seqmatch_intro.jsp (RDP Data—release11_1; Seqmatch—version 3)). The search options enabled were type and non-type strains, environmental sequences and isolates, near full length sequences (≥ 1200) and good quality sequences. The identification of the bacterial isolates was determined on the basis of the highest S_ab score [44].

### Potential bacterial reductase screening

Media (CNM or L broth (LB)) was added to 130.95 mg purified glucoraphanin extract (Intertek Group plc) to a total volume of 2 ml, to generate a 0.15 M glucoraphanin stock solution. Five mg powdered SMCSO (Sigma Aldrich) had 0.22 μm filtered water added to a total of 5 ml to generate a 7.4 mM stock solution. Media (955 μl) had 40 μl 0.15 M glucoraphanin stock solution added to generate a 6 mM glucoraphanin media and CNM (955 μl) and 40 μl 7.4 mM SMCSO stock solution were combined to generate an SMCSO media (0.3 mM). Each were inoculated with 5 μl of bacterial glycerol stocks. *E. coli* DH5α was kindly donated by Dr Fatma Cebeci. Control samples were generated through the addition of 5 μl 0.22 μm filtered water in place of 5 μl bacterial glycerol stock. Samples were incubated under static aerobic or anaerobic conditions at 37˚C for 24 h or 72 h. Optical density readings were taken at 600 nm wavelength using a 6715 UV/vis. spectrophotometer (Jenway) with control samples used as blanks. Samples were centrifuged at 21,100×*g* for 5 min and the supernatant was filter sterilised and stored at − 20 °C for desulphoglucosinolate analysis using HPLC, or SMCSO derivative analysis using LC/MS and ^1^H NMR spectroscopy analysis.

### *S*-methylcysteine sulphoxide analysis

The cultured samples (100 μl) were mixed with 250 μl dansyl chloride reagent (10 mM) and 650 μl borate buffer (20 mM; pH 9.2) and incubated at room temperature for 30 min, prior to centrifugation at 17,000×*g* for 10 min. The supernatants (~ 800 μl) were transferred to autosampler vials and LC–MS analysis was performed with a Waters Spherisorb ODS2 (4.6 × 250 mm id, 5 μm particle size) column connected to an 1100 series Single Quad LC–MS system (Agilent Technologies) comprised of a 1200 series degasser, binary pump, cooled autosampler, column oven, diode array detector and G1956B mass spectrometer. The samples were eluted at 0.9 ml/min with a gradient of increasing acidified methanol (MeOH): solvent A (50 mM ammonium acetate adjusted to pH 5 with 0.1 M acetic acid), solvent B (MeOH (1 l) containing 10 mM hydrochloric acid (826 μl 37% conc.)). The gradient increased from 30% acidified MeOH to 40% over 35 min, then increased from 40 to 75% over 25 min and after 10 min this re-equilibrated to 30% acidified MeOH for 5 min. The LC eluent flow was sprayed into the mass spectrometer interface without splitting. SMCSO isomers were monitored by MS using full scan acquisition with electrospray ionisation source in the positive ion mode. Identification was achieved on the basis of retention time and quantification was performed through the use of calibration standards.

### Data analysis

Data analysis was performed using GraphPad Prism 5.04 unless otherwise stated. A probability of *p* ≤ 0.05 was considered statistically significant. Statistical analysis of lactate levels was performed using one-way ANOVA followed by Bonferroni multiple comparisons tests. Glucosinolate data was statistically analysed using paired Student’s *t* tests (two-tailed). The data for the ratios of glucoraphanin and glucoerucin between bacterial pure culture samples were statistically analysed using two-way ANOVA, whilst the analysis of total glucosinolates in pure culture samples vs the associated control sample were analysed using Student’s *t* tests (two-tailed).

## Results

### Effects of broccoli leachate on faecal microbiome composition

**Fig. 1:**
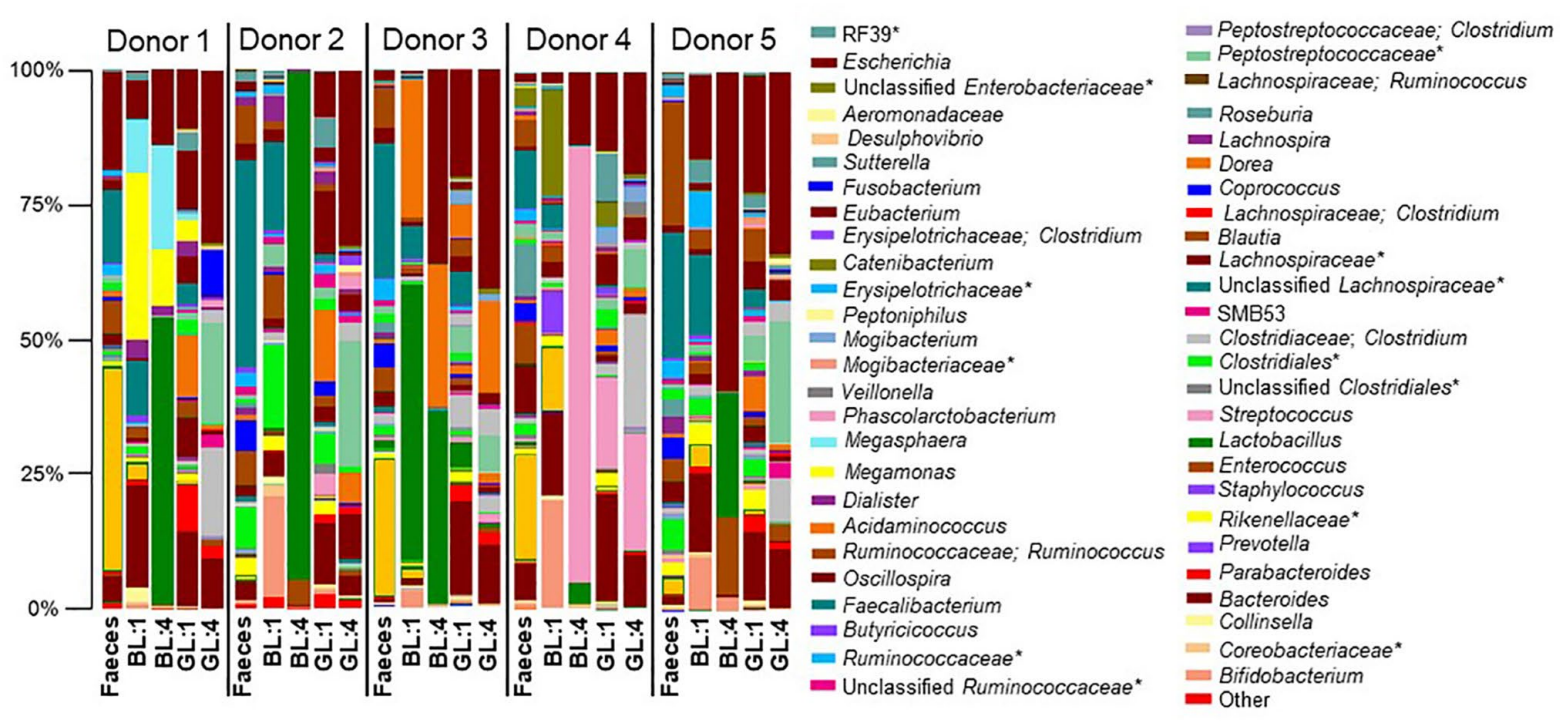
The proportions of *Lactobacillus* or *Streptococcus* increase after culturing faecal microbiomes in a media containing a broccoli leachate. Five human faecal slurries were cultured independently in a broccoli leachate (BL) containing media and a glucose (GL) media. The bar chart displays the proportion of OTUs per cultured faecal microbiome of each donor after the first (1) and last 12 h cycle (4). The key reads from top left to bottom right and consists of genera with a relative abundance ≥ 1% in at least one sample. Asterisk represents OTU reported at a higher taxonomic level

Faecal samples from five healthy donors were used to inoculate both a broccoli leachate-containing (BL) media and a glucose (GL) media in five independent experiments. The cultures were grown anaerobically at 37 °C and an aliquot was taken every 12 h to inoculate the respective fresh media, with further aliquots taken for analyses. This was repeated with four technical replicates for four 12 h cycles, as preliminary work (not shown) indicated these were sufficient parameters to observe stable microbiome modifications. As expected, differences in the initial composition of the faecal microbiome were observed between the five donors (Fig. 1).

As the experiments progressed, a change in microbiome composition occurred and this was largely media-dependent. By the fourth 12 h cycle, relatively large increases in the proportion of *Lactobacillus* was observed in the BL media to varying degrees, but not the GL media (dark green bar in Fig. 1). The average proportion of *Lactobacillus* present in the initial faecal samples was 0.5% (SD ± 0.7%) (Online Resource Table 1). The average proportion of the 5 donors increased to 10.2% (SD ± 20.6%) after cycle 1 and 42.0% (SD ± 31.4%) after cycle 4 in the BL media. As *Lactobacillus* was only found at detectable levels in the GL media at cycle 4 for one donor (donor 3: 0.8% (SD ± 0.1%)), this indicates that the increased growth was due to constituents of the broccoli leachate, rather than the experimental conditions or basal media. Interestingly, although proportional increases of *Lactobacillus* in the BL medium was observed in the cultured microbiome of donor 4 at cycle 4, the community was dominated by another lactic acid bacterium, *Streptococcus* (light pink bar in Fig. 1). The starting media had a pH of 7.0 ± 0. pH measurements taken at the end of cycles 1 and 4 for donors 2–5 indicated the average pH of the inoculated BL media was reduced to 4.5 ± 0.2 and 4.9 ± 0.4 at the end of cycles 1 and 4, respectively (Online Resource Table 2). Conversely, the pH of the cultured GL media remained stable throughout the experiments; 7.1 ± 0.1 and 7.0 ± 0.2 at the end of cycles 1 and 4, respectively.

### Effects of broccoli leachate on bacterial metabolism

**Fig. 2:**
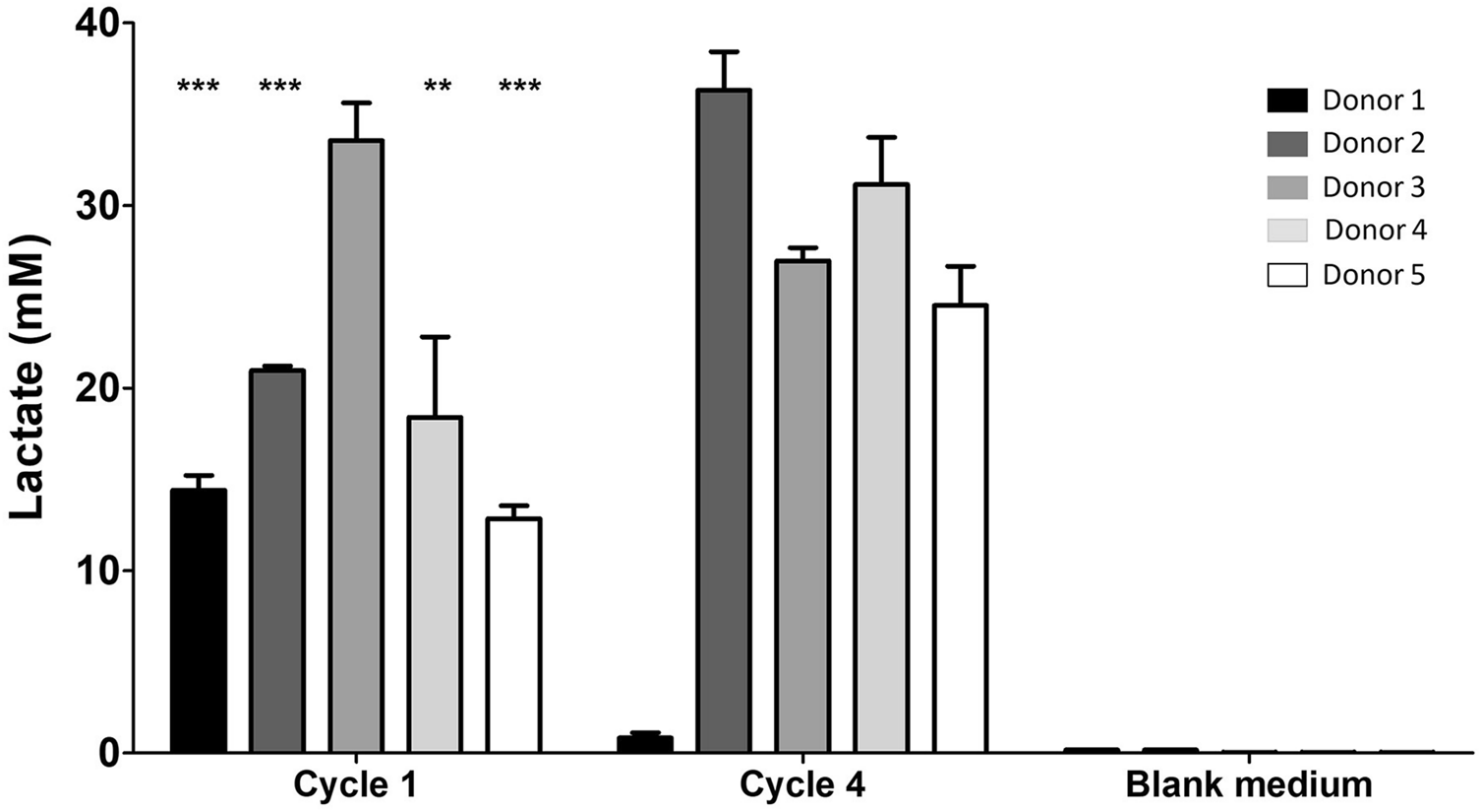
Lactate accumulated in a broccoli leachate-containing media when cultured with human faecal microbiomes. Lactate levels in the pre-inoculated BL media and cultured BL media after cycles 1 and 4 for the microbiomes obtained from donors 1–5. Data shown = mean ± SD of four technical replicates. **p ≤ 0.01, ***p ≤ 0.001 Cycle 1 vs Cycle 4

^1^H NMR spectroscopy quantification of fermented samples showed relatively high levels of lactate (23.5–36.3 mM) for four of the five microbiomes (donors 2–5), in the BL media after cycle 4. However, significantly lower levels of lactate were detected in the BL media at cycle 4 for the cultured microbiome of donor 1 (Fig. 2). The decrease in lactate concentration between cycles 1 and 4 for donor 1 may have been due to increased lactate utilisation by the microbiome. Although lactate was detected in the GL media after cycle 1, when cultured with three of the faecal microbiomes (donors 3–5), it was only present in low amounts (0.07–0.84 mM) (Online Resource table 3). Lactate was only detected in the GL media after cycle 4 when cultured with the faecal microbiome of donor 5 (0.17 mM). ^1^H NMR spectroscopy was used to identify the concentrations of SCFAs (acetate, butyrate, formate, propionate and valerate) in the cultured BL and GL media (Table 2). The inoculated microbiome of donor 1 produced a variety of SCFAs, as well as the highest total yield of SCFAs (60.3 mM). Valerate was only detected in the samples from donors 1 and 3, whilst neither butyrate nor propionate were observed in the cultured samples from donors 2 and 5. The relatively high concentration of valerate in the samples inoculated with the faecal bacteria of donor 1 may be linked to the detection of *Megasphaera*, a known valerate producer, in the cultured bacterial community. The lowest total yield of SCFAs was observed in the sample derived from donor 4 (6.5 mM) and this was largely composed of acetate and formate. On average, across all five faecal microbiomes tested, there was a 2.2-fold increase in total yield of SCFAs in the BL vessels compared to the GL vessels. The increased levels of the SCFAs found in the BL fermentation likely reflects the availability of nutrients derived from the broccoli leachate and will link to the metabolic capabilities of each microbiome.

**Table 2:** Mean short-chain fatty acid concentrations (± SD) in the broccoli leachate (BL) and glucose (GL) media after cycle 4, when cultured with human faecal microbiomes

SCFAs (mM)	Donor 1		Donor 2		Donor 3		Donor 4		Donor 5	
	BL media	GL media	BL media	GL media	BL media	GL media	BL media	GL media	BL media	GL media
Acetate	21.4 (± 0.3)	10.6 (± 2.4)	20.3 (± 1.5)	8.7 (± 0.2)	24.7 (± 7.2)	7.5 (± 1.2)	3.8 (± 2.8)	7.5 (± 0.1)	16.5 (± 2.7)	6.7 (± 1.1)
Butyrate	8.6 (± 0.3)	1.6 (± 0.5)	0	1.7 (± 0.1)	4.9 (± 1.3)	0.82 (± 0.2)	0	1.6 (± 0)	0	0.85 (± 0.3)
Formate	2.3 (± 0.1)	1.9 (± 1.2)	1.2 (± 0.1)	2.2 (± 0.1)	5.0 (± 0.7)	3.0 (± 0.2)	2.6 (± 2.2)	2.4 (± 0.1)	1.7 (± 0.1)	2.5 (± 0.4)
Propionate	16.5 (± 1.1)	2.3 (± 1.2)	0	0.42 (± 0.5)	2.6 (± 2.8)	0.03 (± 0.1)	0.06 (± 0.1)	1.8 (± 0.2)	0	0.88 (± 0.6)
Valerate	11.5 (± 0.3)	0.57 (± 0.3)	0	0.09 (± 0.1)	0.21 (± 0.4)	0.44 (± 0.6)	0	0.16 (± 0.3)	0	0.18 (± 0.1)
Total	60.3	17.0	21.5	13.1	37.4	11.8	6.5	13.5	18.2	11.1

### Reduction of glucosinolates by faecal microbiome

**Fig. 3:**
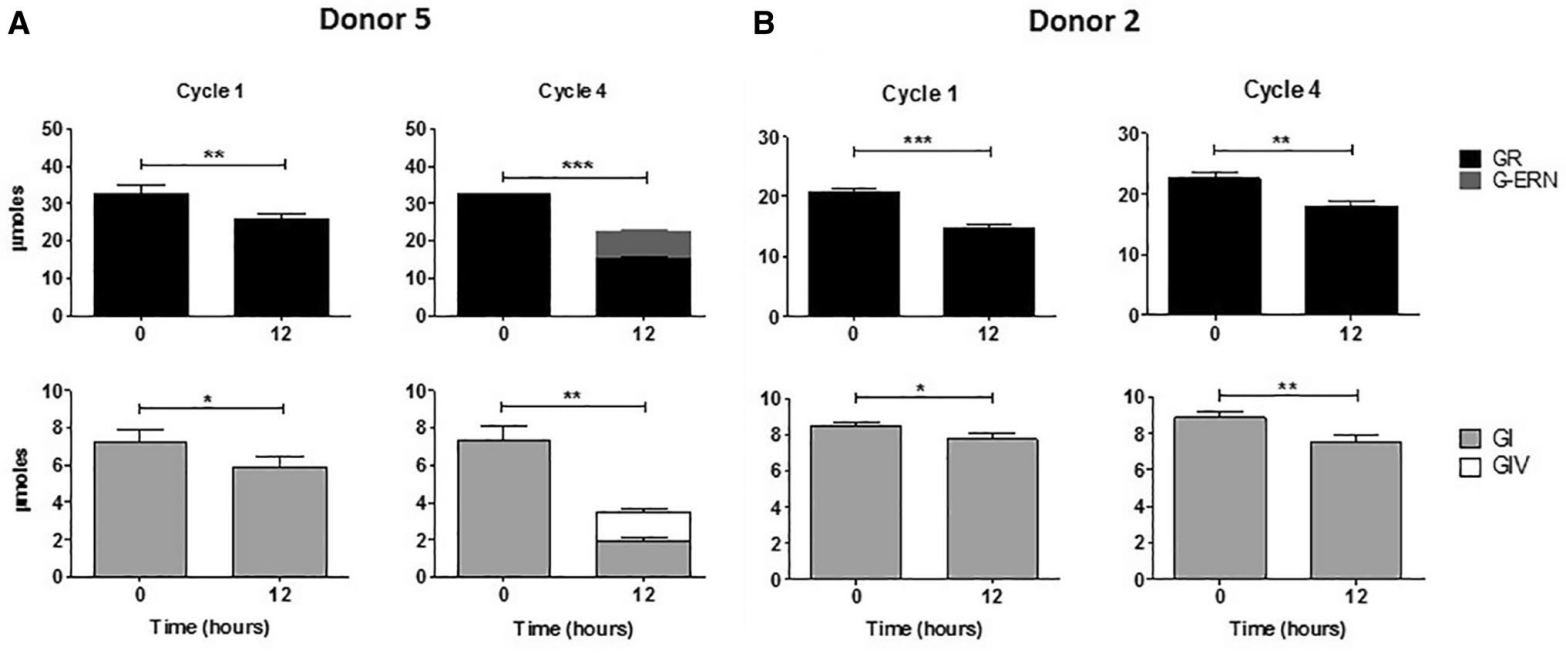
Conversion of glucosinolates to their reduced analogues when cultured with a human faecal microbiome. The conversion of glucoraphanin to glucoerucin and glucoiberin to glucoiberverin by the faecal microbiome of donor 5, which is representative of the trend seen for the microbiomes of donors 1, 3 and 4. No conversion was observed for the microbiome of donor 2. GR; Glucoraphanin: G-ERN; Glucoerucin: GI; Glucoiberin: GIV; Glucoiberverin. Data shown = mean ± SD of four technical replicates. *p ≤ 0.05, **p ≤ 0.01, ***p ≤ 0.001 zero hr vs 12 h

Fermentation samples were also analysed to determine whether these microbial communities were able to metabolise the glucosinolates derived from the broccoli. HPLC analysis indicated that, of five independent experiments, four cultured faecal bacterial communities (donors 1, 3, 4 and 5) exhibited the ability, to varying degrees, to biotransform glucoraphanin and glucoiberin, converting them to their reduced analogues glucoerucin and glucoiberverin, respectively. Figure 3 shows the level of glucosinolate conversion performed by the faecal microbiome of donor 5. However, the bacterial community of donor 2 did not seem to have the ability to reduce the glucosinolates, but as with all the faecal microbiomes tested, a decrease in glucoraphanin and glucoiberin levels were observed across each 12 h cycle (Online Resource table 4).

In order to determine the fate of the missing glucoraphanin at cycle 4, LC–MS/MS was used to identify known glucoraphanin breakdown products. Low levels of the ITC sulphoraphane (SF) and SF nitrile were present in the starting media and the levels of both decreased within each 12 h cycle during all five experiments (Online Resource Fig. 1). However, combining the HPLC and LC–MS/MS data of glucoraphanin, glucoerucin and their related hydrolysis products over each 12 h culturing period, did not account for the total amount of glucoraphanin and associated breakdown products present in the starting media.

**Fig. 4:**
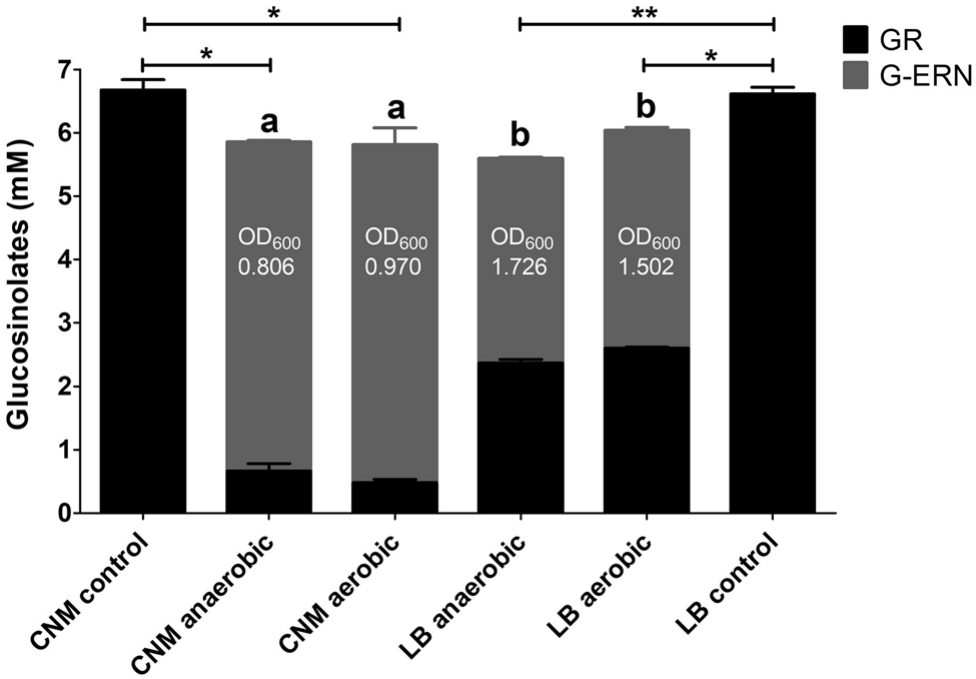
The ratio of glucoraphanin to glucoerucin differs between the CNM and LB media when cultured with *Escherichia coli* 1B04, irrespective of oxygen levels. *E. coli* 1B04 was cultured in CNM and LB media containing purified glucoraphanin extract at ~ 6 mM for 24 h at 37 °C, under both anaerobic and aerobic conditions. GR, glucoraphanin; G-ERN, glucoerucin; CNM, chemostat nutrient media; LB , L broth. Data shown = mean ± SD of two technical replicates. Ratio of glucoraphanin and glucoerucin between samples; **a** vs **b** *p* ≤ 0.001. Total glucosinolates in cultured samples vs control sample; *p ≤ 0.05, **p ≤ 0.01

Ten bacterial isolates, obtained from BL media cultured microbiomes, were selected at random and screened for their ability to convert glucoraphanin to glucoerucin in pure cultures. Four isolates identified as belonging to the genus *Escherichia* were able to substantially reduce glucoraphanin to glucoerucin (77.7–89%) (Online Resource table 5). The remaining six isolates, that did not exhibit the ability to reduce glucoraphanin, included bacteria from the order *Lactobacillales*: four *L. fermentum* isolates and two *Enterococcus* species (*E. faecium* and *E. durans*). One *E. coli* (strain 1B04) with the ability to reduce glucoraphanin to glucoerucin was chosen for further study. This isolate was cultured separately in CNM and LB supplemented with purified glucoraphanin to a concentration of ~ 6 mM under aerobic and anaerobic conditions to investigate whether differences in atmosphere or media would affect bacterial reductase activity. No significant difference in reductase activity was observed between aerobic or anaerobic conditions within each media type, but the conversion of glucoraphanin to glucoerucin was lower in LB media, compared to CNM media (Fig. 4). However, when the culturing period was extended to 72 h under anaerobic conditions (aerobic conditions not tested), a similar level of glucoraphanin conversion was observed for both media types, although the optical density measurements at 72 h were similar to those recorded at 24 h for each cultured media; CNM—0.798, LB—1.400 (Online Resource Fig. 2).

**Fig. 5:**
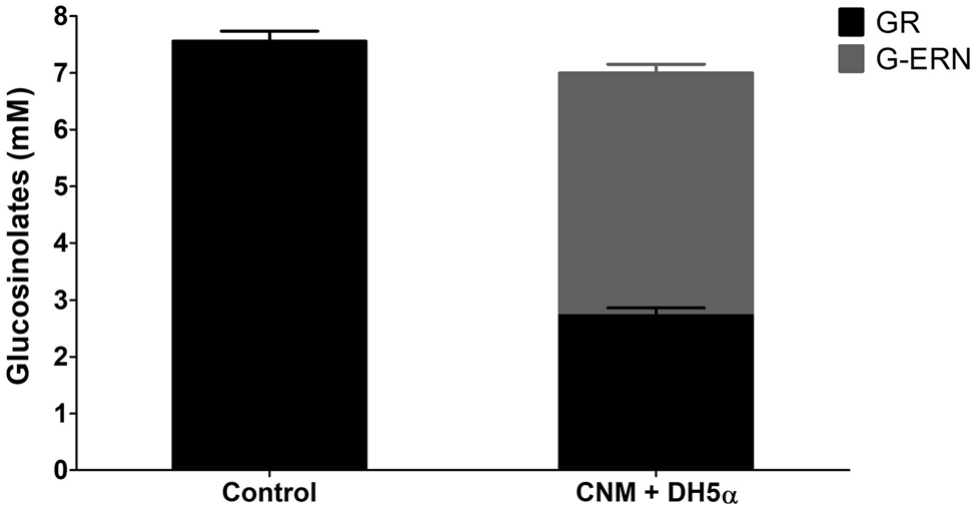
The laboratory strain, *Escherichia coli* DH5α, has the ability to convert glucoraphanin to glucoerucin. *E. coli* DH5α was cultured in CNM containing ~ 7 mM purified glucoraphanin extract for 24 h at 37 °C under anaerobic conditions. GR = Glucoraphanin; G-ERN = Glucoerucin; CNM = Chemostat nutrient media; DH5α = *Escherichia coli* DH5α. Data shown = mean ± SD of three technical replicates

Further work with *E. coli* 1B04 indicated that this bacterial isolate was unable to convert glucoerucin to glucoraphanin via an oxidation reaction, or hydrolyse glucoerucin (Online Resource Fig. 3). We also tested the laboratory strain *E. coli* DH5α, which was developed as a laboratory cloning strain via mutagenesis of an isolate obtained from a patient in 1920 [45]. *E. coli* DH5α was cultured in CNM containing glucoraphanin to assess whether the ability to reduce glucoraphanin was specifically associated with *E. coli* adapted to the human gut, as DH5α is considered to not have a natural habitat*.* Although *E. coli* DH5α grew relatively poorly in this media (OD_600_ = 0.286), this strain was still able to convert glucoraphanin to glucoerucin (Fig. 5). This suggests that the ability to reduce sulphoxide groups is not restricted to gut-adapted *E. coli* and may be a general metabolic process rather than a glucosinolate-specific process.

### Bacterial reduction of *S*-methylcysteine sulphoxide (SMCSO)

**Fig. 6:**
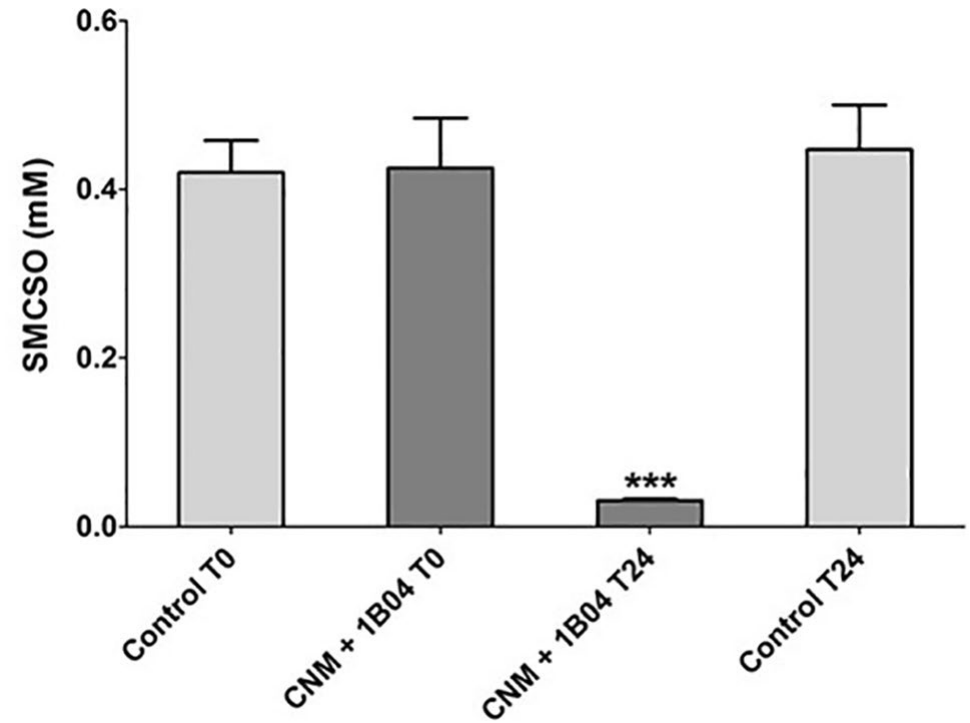
Levels of *S*-methylcysteine sulphoxide decrease when incubated with *E. coli* 1B04. *E. coli* 1B04 was cultured in CNM containing 0.3 mM SMCSO for 24 h at 37 °C, under anaerobic conditions. SMCSO = *S*-methylcysteine sulphoxide; CNM = Chemostat nutrient media; 1B04 = *Escherichia coli* 1B04; T0 = prior to inoculation; T24 = 24 h post-inoculation. Data shown = mean ± SD of three technical replicates

**Fig. 7:**
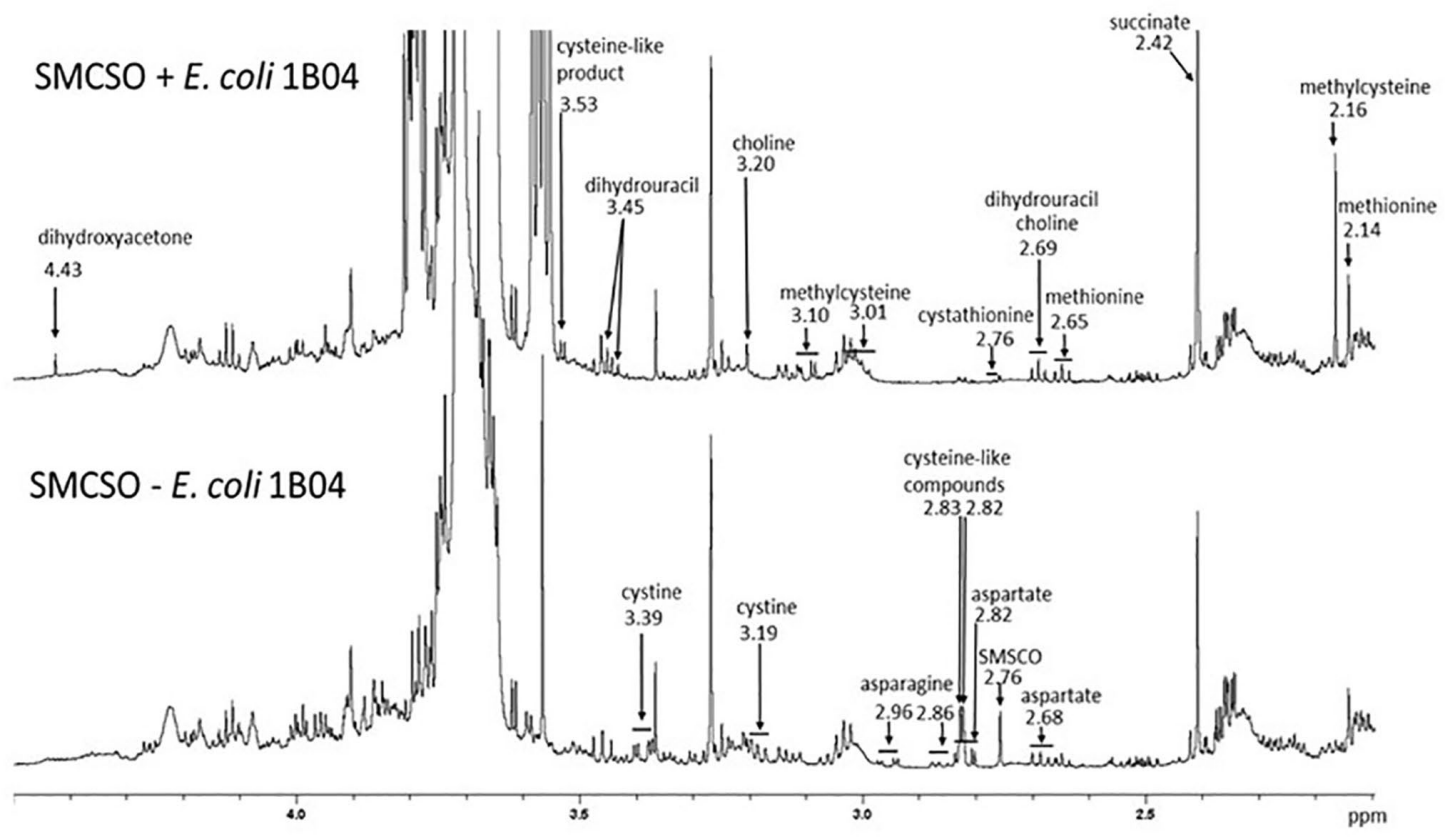
^1^H NMR spectra of medium supplemented with *S*-methylcysteine sulphoxide with and without *E. coli* 1B04. Substrates, such as SMSCO, aspartate, asparagine, cystine, proline, trehalose and some nucleosides (not shown) were consumed. Products, such as methylcysteine, cystathionine (another cysteine derivate), methionine, choline, dihydroxyacetone, dihydrouracil, succinate, fumarate, ethanol, acetate and formate (not shown) appeared

To investigate whether the observed putative reductase activity was glucoraphanin-specific or extendable to other compounds, *E. coli* 1B04 was cultured in a media containing SMCSO, another broccoli-derived compound containing a sulphoxide moiety. Levels of SMCSO decreased significantly over a period of 24 h in the presence of *E. coli* 1B04 (Fig. 6). SMCSO levels remained stable in the control samples devoid of bacteria, reflecting the importance of *E. coli* 1B04 for the metabolic breakdown of SMCSO. Analysis using ^1^H NMR spectroscopy identified *S*-methylcysteine, the reduced analogue of SMCSO, which was only detected in the SMCSO-containing CNM media incubated with *E. coli* 1B04 (Fig. 7).

## Discussion

The current study demonstrated that culturing human faecal microbiomes in the presence of broccoli leachate (BL) resulted in an alteration of the composition of the in vitro faecal microbiome. After the fourth cycle of culturing the microbiome in BL, the microbiomes of 4 of the 5 donors were dominated by *Lactobacillus*, whereas this modification was not observed when the microbiome was cultured in media containing glucose. In a previous study by Filannino et al. [46], the metabolism of phenolic compounds by *Lactobacillus* sp. was investigated using a broccoli puree. All strains tested exhibited good growth and it was found that two strains of *Lactobacillus* (*L. fermentum* FUA3165 and *L. reuteri* FUA3168) were able to reduce quinic acid, which was postulated to give these organisms an energetic advantage through NAD^+^ regeneration [46]. In vitro and in vivo studies have indicated that the bacterial metabolism of dietary components, such as fibres, polyphenols and glycated proteins, may have the potential to modify gut microbiomes via an increase of beneficial intestinal lactobacilli [47–50]. In this study, the increase in lactobacilli is likely largely due to the presence of sugars, however soluble fibre may have also played a role but fibre analyses were not carried out so this cannot be confirmed.

In one experiment, with an independent donor, where a bloom of *Lactobacillus* was not observed, *Streptococcus* was present at the largest proportion. *Lactobacillus* and *Streptococcus* are from the order *Lactobacillales* and are considered core members of the LAB. This group of Gram-positive bacteria are found in the small intestine and colon, where they metabolise dietary carbohydrates. Broccoli is rich in galacturonic acid, glucose, galactose and arabinose, with lesser amounts of mannose, xylose, rhamnose and fucose also present [51]. This source of carbohydrates may have provided *Lactobacillus* and *Streptococcus* with a competitive advantage over other members of the in vitro microbiome. The observed proportional increase in LAB was correlated with a decrease in the pH of the fermentation media from 7.0 to 4.9. However, it is important to note that the bacterial metabolism of simpler carbohydrates, such as sugars, largely takes place in the small intestine and the pH decreases observed in these experiments do not reflect the pH of the human colon. Therefore, although the microbiome results are of interest in an in vitro setting, we would not expect to observe similar compositional changes in vivo. ^1^H NMR spectroscopy of the culture media indicate that the observed decrease in pH was associated with the production of lactic acid by the LAB. However, in one fermentation with the microbiome of donor 1 the levels of lactate did not increase. Lactate is the major metabolic end product of carbohydrate fermentation by LAB and can be subsequently utilised by other members of the gut microbiome to generate bacterial metabolites, such as SCFAs. Acetate, butyrate, formate, propionate and valerate were all detected during the fermentation of both the broccoli leachate or glucose, but there was on average, a 2.2-fold increase in the total yield of these SCFAs in the presence of BL. We hypothesised that the microbiomes cultured in the BL media utilised available lactate for metabolic processes, leading to a greater yield of SCFAs.

Acetate was the primary SCFA produced from all cultured microbiomes, as has been observed in the human colon and faeces [52]. Acetate or butyrate can be produced through the bacterial utilisation of pyruvate, which can be formed through the oxidation of lactate [53], or lactate can be used directly in the acrylate pathway to generate propionate [54]. Relatively high concentrations of propionate were only detected in the fermentations with BL (Table 2). This may indicate that lactate was being produced by the microbiome, but was partly channelled into the production of propionate. The microbiome of donor 1 contained a relatively high proportion of the genus *Megasphaera*, which was not detected in the other samples. *Megasphaera elsdenii* is an intestinal bacterium that converts lactate to propionate via the acrylate pathway [55]. When glucose and lactate are both available, *M. elsdenii* produces increasing amounts of butyrate and valerate, with a concomitant decrease in the production of propionate [56]. When glucose is depleted, lactate is metabolised to produce propionate. The presence of *Megasphaera* in the BL media cultured with the faecal microbiome of donor 1 may be at least partly responsible for the relatively high concentration of both propionate and valerate, as well as contributing to butyrate production. SCFAs are mainly produced by the bacterial fermentation of non-digestible carbohydrates that pass through the small intestine to the colon. However, under the in vitro conditions detailed here, the bacteria would be able to use the available sugars to produce SCFAs. These results would have been strengthened by analysing the soluble fibre content of the BL media and as such it is difficult to relate these results to in vivo SCFA production following the consumption of broccoli.

When human faecal bacteria were cultured in a BL media, decreases in the levels of glucoraphanin and glucoiberin were recorded for each experiment, with a concomitant appearance of the reduced analogues glucoerucin and glucoiberverin, respectively, observed in four of the five experiments. This reduction was microbiome dependent and the reduction of glucosinolates by a human faecal microbiome [25] and bacteria isolated from human faecal material [23] has previously been reported. This suggests that the microbiome of donor 2, in which glucosinolate reduction was not observed, may have lacked the bacteria able to perform the reduction reaction.

We also examined the ability of individual gut bacteria to reduce glucoraphanin.

We identified four strains with this activity, all of which belong to the genus *Escherichia*. It is interesting to note that the BL fermentation with the faecal microbiome of donor 2 did not show a high abundance of *Escherichia* and the conversion of glucoraphanin to glucoerucin was not observed. Whilst the anaerobic environment did not play a role in the reduction of glucoraphanin by *E. coli* 1B04, the type of culture media used affected the rate of glucoraphanin reduction, with lower levels of glucoerucin detected in LB, compared to CNM, over 24 h. This corresponded to increased growth of *E. coli* 1B04 in LB, compared to CNM. Differences in potential co-factors between the media may also impact on this reduction activity.

Growth of *E. coli* 1B04 in CNM media containing glucoerucin indicated that glucoerucin was not metabolised. Research by Simala–Grant and Weiner showed that the anaerobic growth of *E. coli*, on various sulphoxides, was only supported following the reduction of these compounds by DMSO reductase [57]. This enabled the sulphoxides to act as electron acceptors, which can be used by *E. coli* to drive ATP synthesis through anaerobic respiration. Therefore, the difference in the rate of glucoraphanin reduction observed in this study may reflect the presence of preferred electron acceptors in the LB media, which are modified by other enzymes. *E. coli* is known to express a wide range of reductases, such as terminal reductases used in anaerobic respiration [58], methionine sulphoxide reductases that counteract damage that can be caused by reactive oxygen species [59] and ribonucleotide reductases, which are involved in the synthesis of DNA [60].

The laboratory strain *E. coli* DH5α was also able to reduce glucoraphanin, suggesting that the enzymes responsible for the glucosinolate reduction may be broad-range non-specific enzymes. It is not unreasonable to suggest that sulphoxide reduction in anaerobic environments may provide electron acceptors for anaerobic respiration, whilst similar reductase activity in an aerobic environment may serve to protect *E. coli* from oxidative damage. To test the hypothesis that the observed glucoraphanin reduction was not glucosinolate-specific, *E. coli* 1B04 was cultured in CNM containing *S*-methylcysteine sulphoxide (SMCSO). A significant decrease in the levels of SMCSO was observed and ^1^H NMR analysis indicated that a large proportion of SMCSO was putatively converted to the reduced analogue, *S*-methylcysteine. Although glucoraphanin and SMCSO may have been reduced by the actions of different enzymes, it is plausible that the activity of a single enzyme with broad substrate specificity is responsible.

This study reinforces the point that the composition of bacterial communities and the profile of their metabolites can be directly affected by diet in general. However, the bioconversion of individual phytochemicals within the diet, such as glucosinolates, may confer additional health benefits to the host. We believe that this is the first study that shows the reduction of the dietary compound *S*-methylcysteine sulphoxide by bacteria isolated from human faeces.

## Electronic supplementary material

Below is the link to the electronic supplementary material.

Supplementary file1 (DOCX 256 kb)

## References

[1] Dominguez-Bello MG, Costello EK, Contreras M, Magris M, Hidalgo G, Fierer N, Knight R (2010). Delivery mode shapes the acquisition and structure of the initial microbiota across multiple body habitats in newborns. Proc Natl Acad Sci USA.

[2] Claesson MJ, Cusack S, O'Sullivan O, Greene-Diniz R, de Weerd H, Flannery E, Marchesi JR, Falush D, Dinan T, Fitzgerald G, Stanton C, van Sinderen D, O'Connor M, Harnedy N, O'Connor K, Henry C, O'Mahony D, Fitzgerald AP, Shanahan F, Twomey C, Hill C, Ross RP, O'Toole PW (2011). Composition, variability, and temporal stability of the intestinal microbiota of the elderly. Proc Natl Acad Sci USA.

[3] Goodrich JK, Waters JL, Poole AC, Sutter JL, Koren O, Blekhman R, Beaumont M, Van Treuren W, Knight R, Bell JT, Spector TD, Clark AG, Ley RE (2014). Human genetics shape the gut microbiome. Cell.

[4] Dethlefsen L, Relman DA (2011). Incomplete recovery and individualized responses of the human distal gut microbiota to repeated antibiotic perturbation. Proc Natl Acad Sci USA.

[5] Yatsunenko T, Rey FE, Manary MJ, Trehan I, Dominguez-Bello MG, Contreras M, Magris M, Hidalgo G, Baldassano RN, Anokhin AP, Heath AC, Warner B, Reeder J, Kuczynski J, Caporaso JG, Lozupone CA, Lauber C, Clemente JC, Knights D, Knight R, Gordon JI (2012). Human gut microbiome viewed across age and geography. Nature.

[6] David LA, Maurice CF, Carmody RN, Gootenberg DB, Button JE, Wolfe BE, Ling AV, Devlin AS, Varma Y, Fischbach MA, Biddinger SB, Dutton RJ, Turnbaugh PJ (2014). Diet rapidly and reproducibly alters the human gut microbiome. Nature.

[7] De Filippo C, Cavalieri D, Di Paola M, Ramazzotti M, Poullet JB, Massart S, Collini S, Pieraccini G, Lionetti P (2010). Impact of diet in shaping gut microbiota revealed by a comparative study in children from Europe and rural Africa. Proc Natl Acad Sci USA.

[8] Ley RE, Peterson DA, Gordon JI (2006). Ecological and evolutionary forces shaping microbial diversity in the human intestine. Cell.

[9] Turnbaugh PJ, Hamady M, Yatsunenko T, Cantarel BL, Duncan A, Ley RE, Sogin ML, Jones WJ, Roe BA, Affourtit JP, Egholm M, Henrissat B, Heath AC, Knight R, Gordon JI (2009). A core gut microbiome in obese and lean twins. Nature.

[10] Wu GD, Chen J, Hoffmann C, Bittinger K, Chen Y-Y, Keilbaugh SA, Bewtra M, Knights D, Walters WA, Knight R, Sinha R, Gilroy E, Gupta K, Baldassano R, Nessel L, Li H, Bushman FD, Lewis JD (2011). Linking long-term dietary patterns with gut microbial enterotypes. Science.

[11] Masood MI, Qadir MI, Shirazi JH, Khan IU (2011). Beneficial effects of lactic acid bacteria on human beings. Crit Rev Microbiol.

[12] Macfarlane S, Macfarlane GT (2003). Regulation of short-chain fatty acid production. Proc Nutr Soc.

[13] Nicholson JK, Wilson ID (2003). Understanding 'global' systems biology: Metabonomics and the continuum of metabolism. Nat Rev Drug Discovery.

[14] Duncan SH, Louis P, Thomson JM, Flint HJ (2009). The role of pH in determining the species composition of the human colonic microbiota. Environ Microbiol.

[15] Podsedek A (2007). Natural antioxidants and antioxidant capacity of Brassica vegetables: a review. Lwt Food Sci Technol.

[16] Cheng DL, Hashimoto K, Uda Y (2004). In vitro digestion of sinigrin and glucotropaeolin by single strains of Bifidobacterium and identification of the digestive products. Food Chem Toxicol.

[17] Elfoul L, Rabot S, Khelifa N, Quinsac A, Duguay A, Rimbault A (2001). Formation of allyl isothiocyanate from sinigrin in the digestive tract of rats monoassociated with a human colonic strain of Bacteroides thetaiotaomicron. FEMS Microbiol Lett.

[18] Palop ML, Smiths JP, Tenbrink B (1995). Degradation of sinigrin by lactobacillus-agilis strain R16. Int J Food Microbiol.

[19] Oginsky EL, Stein AE, Greer MA (1965). Myrosinase activity in bacteria as demonstrated by conversion of progoitrin to goitrin. Proc Soc Exp Biol Med.

[20] Bheemreddy RM, Jeffery EH (2007). The metabolic fate of purified glucoraphanin in F344 rats. J Agric Food Chem.

[21] Shapiro TA, Fahey JW, Wade KL, Stephenson KK, Talalay P (1998). Human metabolism and excretion of cancer chemoprotective glucosinolates and isothiocyanates of cruciferous vegetables. Cancer Epidemiol Biomark Prev.

[22] Li F, Hullar MAJ, Beresford SAA, Lampe JW (2011). Variation of glucoraphanin metabolism in vivo and ex vivo by human gut bacteria. Br J Nutr.

[23] Luang-In V, Narbad A, Nueno-Palop C, Mithen R, Bennett M, Rossiter JT (2014). The metabolism of methylsulfinylalkyl- and methylthioalkyl-glucosinolates by a selection of human gut bacteria. Mol Nutr Food Res.

[24] Narbad A, Rossiter JT (2018). Gut glucosinolate metabolism and isothiocyanate production. Mol Nutr Food Res.

[25] Saha S, Hollands W, Teucher B, Needs PW, Narbad A, Ortori CA, Barrett DA, Rossiter JT, Mithen RF, Kroon PA (2012). Isothiocyanate concentrations and interconversion of sulforaphane to erucin in human subjects after consumption of commercial frozen broccoli compared to fresh broccoli. Mol Nutr Food Res.

[26] Edmands WMB, Gooderham NJ, Holmes E, Mitchell SC (2013). S-Methyl-L-cysteine sulphoxide: the Cinderella phytochemical?. Toxicol Res.

[27] Hamamoto A, Mazelis M (1986). THE C-S lyases of higher-plants - isolation and properties of homogeneous Cystine lyase from broccoli (*Brassica-oleracea* var botrytis) buds. Plant Physiol.

[28] Larsen GL (1985). Distribution of cysteine conjugate beta-lyase in gastrointestinal bacteria and in the environment. Xenobiotica.

[29] Nomura J, Nishizuka Y, Hayaishi O (1963). S-alkylcysteinase—enzymatic cleavage of S-methyl-L-cysteine and its sulfoxide. J Biol Chem.

[30] Kim S-Y, Park K-W, Kim J-Y, Jeong I-Y, Byun M-W, Park J-E, Yee S-T, Kim K-H, Rhim JS, Yamada K, Seo K-I (2008). Thiosulfinates from *Allium tuberosum* L. induce apoptosis via caspase-dependent and -independent pathways in PC-3 human prostate cancer cells. Bioorg Med Chem Lett.

[31] Marks HS, Anderson JA, Stoewsand GS (1993). Effect of S-methyl cysteine sulfoxide and its metabolite methyl methane thiosulfinate, both occurring naturally in brassica vegetables, on mouse genotoxicity. Food Chem Toxicol.

[32] Reddy BS, Kawamori T, Lubet R, Steele V, Kelloff G, Rao CV (1999). Chemopreventive effect of S-methylmethane thiosulfonate and sulindac administered together during the promotion/progression stages of colon carcinogenesis. Carcinogenesis.

[33] Kumari K, Augusti KT (2002). Antidiabetic and antioxidant effects of S-methyl cysteine sulfoxide isolated from onions (*Allium cepa* Linn) as compared to standard drugs in alloxan diabetic rats. Indian J Exp Biol.

[34] Kumari K, Augusti KT (2007). Lipid lowering effect of S-methyl cysteine sulfoxide from *Allium cepa* Linn in high cholesterol diet fed rats. J Ethnopharmacol.

[35] Fujiwara M, Uchino H, Inoue K, Itokawa Y (1972). Anti-hypercholesterolemic effect of a sulfur-containing amino-acid, *S*-methyl-l-cysteine sulfoxide, isolated from cabbage. Experientia.

[36] Kellingray L, Tapp HS, Saha S, Doleman JF, Narbad A, Mithen RF (2017). Consumption of a diet rich in Brassica vegetables is associated with a reduced abundance of sulphate-reducing bacteria: a randomised crossover study. Mol Nutr Food Res.

[37] Ellis RJ, Bruce KD, Jenkins C, Stothard JR, Ajarova L, Mugisha L, Viney ME (2013). Comparison of the distal gut microbiota from people and animals in Africa. PLoS ONE.

[38] Caporaso JG, Kuczynski J, Stombaugh J, Bittinger K, Bushman FD, Costello EK, Fierer N, Pena AG, Goodrich JK, Gordon JI, Huttley GA, Kelley ST, Knights D, Koenig JE, Ley RE, Lozupone CA, McDonald D, Muegge BD, Pirrung M, Reeder J, Sevinsky JR, Tumbaugh PJ, Walters WA, Widmann J, Yatsunenko T, Zaneveld J, Knight R (2010). QIIME allows analysis of high-throughput community sequencing data. Nat Methods.

[39] Wang Q, Garrity GM, Tiedje JM, Cole JR (2007). Naive Bayesian classifier for rapid assignment of rRNA sequences into the new bacterial taxonomy. Appl Environ Microbiol.

[40] Le Gall G, Guttula K, Kellingray L, Tett AJ, Ten Hoopen R, Kemsley KE, Savva GM, Ibrahim A, Narbad A (2018). Metabolite quantification of faecal extracts from colorectal cancer patients and healthy controls. Oncotarget.

[41] Magrath R, Bano F, Morgner M, Parkin I, Sharpe A, Lister C, Dean C, Turner J, Lydiate D, Mithen R (1994). Genetics of aliphatic glucosinolates. 1. Side-chain elongation in *Brassica napus* and *Arabidopsis thaliana*. Heredity.

[42] Wang RF, Cao WW, Cerniglia CE (1996). Phylogenetic analysis of *Fusobacterium prausnitzii* based upon the 16S rRNA gene sequence and PCR confirmation. Int J Syst Bacteriol.

[43] Nueno-Palop C, Narbad A (2011). Probiotic assessment of *Enterococcus faecalis* CP58 isolated from human gut. Int J Food Microbiol.

[44] Cole JR, Wang Q, Fish JA, Chai B, McGarrell DM, Sun Y, Brown CT, Porras-Alfaro A, Kuske CR, Tiedje JM (2014). Ribosomal database project: data and tools for high throughput rRNA analysis. Nucleic Acids Res.

[45] Hanahan D (1983). Studies on transformation of *Escherichia coli* with plasmids. J Mol Biol.

[46] Filannino P, Bai Y, Di Cagno R, Gobbetti M, Gaenzle MG (2015). Metabolism of phenolic compounds by *Lactobacillus* spp. during fermentation of cherry juice and broccoli puree. Food Microbiol.

[47] Costabile A, Klinder A, Fava F, Napolitano A, Fogliano V, Leonard C, Gibson GR, Tuohy KM (2008). Whole-grain wheat breakfast cereal has a prebiotic effect on the human gut microbiota: a double-blind, placebo-controlled, crossover study. Br J Nutr.

[48] Costabile A, Kolida S, Klinder A, Gietl E, Baeuerlein M, Frohberg C, Landschuetze V, Gibson GR (2010). A double-blind, placebo-controlled, cross-over study to establish the bifidogenic effect of a very-long-chain inulin extracted from globe artichoke (*Cynara scolymus*) in healthy human subjects. Br J Nutr.

[49] Liu Z, Lin X, Huang G, Zhang W, Rao P, Ni L (2014). Prebiotic effects of almonds and almond skins on intestinal microbiota in healthy adult humans. Anaerobe.

[50] Dominika Ś, Arjan N, Karyn RP, Henryk K (2011). The study on the impact of glycated pea proteins on human intestinal bacteria. Int J Food Microbiol.

[51] Houben K, Jolie RP, Fraeye I, Van Loey AM, Hendrickx ME (2011). Comparative study of the cell wall composition of broccoli, carrot, and tomato: structural characterization of the extractable pectins and hemicelluloses. Carbohyd Res.

[52] Binder HJ (2010) Role of colonic short-chain fatty acid transport in diarrhea. In: Annual review of physiology, vol 72. Annual Review of Physiology. pp 297–313. 10.1146/annurev-physiol-021909-135817 10.1146/annurev-physiol-021909-135817 20148677

[53] Guo T, Zhang L, Xin Y, Xu Z, He H, Kong J (2017). The oxygen-inducible conversion of lactate to acetate in heterofermentative *Lactobacillus brevis* ATCC367. Appl Environ Microbiol.

[54] Reichardt N, Duncan SH, Young P, Belenguer A, Leitch CM, Scott KP, Flint HJ, Louis P (2014). Phylogenetic distribution of three pathways for propionate production within the human gut microbiota. ISME J.

[55] Prabhu R, Altman E, Eiteman MA (2012). Lactate and Acrylate metabolism by *Megasphaera elsdenii* under batch and steady-state conditions. Appl Environ Microbiol.

[56] Marounek M, Fliegrova K, Bartos S (1989). Metabolism and some characteristics of ruminal strains of megasphaera-elsdenii. Appl Environ Microbiol.

[57] SimalaGrant JL, Weiner JH (1996). Kinetic analysis and substrate specificity of *Escherichia coli* dimethyl sulfoxide reductase. Microbiology-Uk.

[58] Unden G, Bongaerts J (1997). Alternative respiratory pathways of *Escherichia coli*: energetics and transcriptional regulation in response to electron acceptors. Biochim Biophys Acta-Bioenergetics.

[59] Ezraty B, Grimaud R, El Hassouni M, Moinier D, Barras F (2004). Methionine sulfoxide reductases protect Ffh from oxidative damages in *Escherichia coli*. EMBO J.

[60] Gon S, Faulkner MJ, Beckwith J (2006). In vivo requirement for glutaredoxins and thioredoxins in the reduction of the ribonucleotide reductases of *Escherichia coli*. Antioxid Redox Signal.

