# Supplementary material for: Effects of in vitro metabolism of a broccoli leachate, glucosinolates and S-methylcysteine sulphoxide on the human faecal microbiome
Source: Eur J Nutr. 2020 Oct 16;60(4):2141–54. doi: 10.1007/s00394-020-02405-y (PMC8137612; doi:10.1007/s00394-020-02405-y)
Supplement: Supplementary file 1 — Supplementary file1 (DOCX 256 kb) [file 394_2020_2405_MOESM1_ESM.docx]

Online Resources

**Effects of *in-vitro* metabolism of broccoli-derived phytochemicals on the structure and function of the human gut microbiome.**

European Journal of Nutrition

Lee Kellingray^a^*, Gwénaëlle Le Gall^b^, Joanne F. Doleman^a^, Arjan Narbad^c^, and Richard F. Mithen^a^.

^a^ Food Innovation and Health, Quadram Institute Bioscience, Norwich Research Park, Norwich, NR4 7UQ, UK.

^b^ Analytical Sciences Unit, Quadram Institute Bioscience, Norwich Research Park, Norwich, NR4 7UQ, UK.

^c^ Gut Microbes and Health, Quadram Institute Bioscience, Norwich Research Park, Norwich, NR4 7UQ, UK.

*Corresponding author: Lee Kellingray (lee.kellingray@quadram.ac.uk)

**Results**

**Online Resource table 1: Percentage of *Lactobacillus* present in the faecal microbial communities throughout the five experiments**

| **Source of microbiome** | **Faeces (%)** | **Broccoli leachate media (%)** | | **Glucose media (%)** | |
| --- | --- | --- | --- | --- | --- |
|  | *Inocula* | *Cycle 1* | *Cycle 4* | *Cycle 1* | *Cycle 4* |
| Donor 1 | 0 | 0 | 53.4 | 0 | 0 |
| Donor 2 | 0 | 0.3 | 94.4 | 0 | 0 |
| Donor 3 | 1.6 | 51.1 | 4.4 | 36.0 | 0.8 |
| Donor 4 | 0.8 | 0.2 | 0.4 | 3.9 | 0 |
| Donor 5 | 0.1 | 0.2 | 0.4 | 23.2 | 0 |

**Online Resource table 2: pH of the cultured faecal microbial communities at cycles 1 & 4 for four of the five experiments.** nk = not known

| **Source of microbiome** | **Broccoli leachate media** | | **Glucose media** | |
| --- | --- | --- | --- | --- |
|  | *Cycle 1* | *Cycle 4* | *Cycle 1* | *Cycle 4* |
| Donor 1 | nk | nk | nk | nk |
| Donor 2 | 4.6 | 5.0 | 7.3 | 7.3 |
| Donor 3 | 4.7 | 5.4 | 7.1 | 6.8 |
| Donor 4 | 4.2 | 4.4 | 7.1 | 7.0 |
| Donor 5 | 4.5 | 4.6 | 7.1 | 7.0 |

**Online Resource table 3: Concentration of lactate present in the faeces and cultured microbial communities throughout the five experiments**

| **Source of microbiome** | **Faeces (mM)** | **Broccoli leachate media (mM)** | | **Glucose media (mM)** | |
| --- | --- | --- | --- | --- | --- |
|  | *Inocula* | *Cycle 1* | *Cycle 4* | *Cycle 1* | *Cycle 4* |
| Donor 1 | 0 | 14.4 | 0.84 | 0 | 0 |
| Donor 2 | 0 | 21.0 | 36.3 | 0 | 0 |
| Donor 3 | 0.02 | 33.4 | 23.5 | 0.35 | 0 |
| Donor 4 | 0.59 | 18.4 | 31.2 | 0.84 | 0 |
| Donor 5 | 0 | 12.8 | 24.5 | 0.07 | 0.17 |

**Online Resource table 4: Levels of glucoraphanin and glucoiberin, and their reduced analogues: glucoerucin and glucoiberverin.** * = No T0 data recorded for donor 1

| **Faecal donor** | **Glucoraphanin (µmoles)** | | | | **Glucoerucin (µmoles)** | | | | **Glucoiberin (µmoles)** | | | | **Glucoiberverin (µmoles)** | | | | |
| --- | --- | --- | --- | --- | --- | --- | --- | --- | --- | --- | --- | --- | --- | --- | --- | --- | --- |
|  | *Cycle 1* | | *Cycle 4* | | *Cycle 1* | | *Cycle 4* | | *Cycle 1* | | *Cycle 4* | | *Cycle 1* | | *Cycle 4* | | |
|  | *T0* | *T12* | *T0* | *T12* | *T0* | *T12* | *T0* | *T12* | *T0* | *T12* | *T0* | *T12* | *T0* | *T12* | *T0* | *T12* |  |
| **1** | * | 29.0  (± 2.2) | * | 9.0  (± 2.9) | * | 0  (± 0) | * | 18.3  (± 0.9) | * | 6.3  (± 0.4) | * | 0.64  (± 0.8) | * | 0  (± 0) | * | 4.3  (± 0.9) |  |
| **2** | 20.7  (± 0.8) | 14.7  (± 0.5) | 22.6  (± 1.0) | 18.0  (± 0.8) | 0  (± 0) | 0  (± 0) | 0  (± 0) | 0  (± 0) | 8.5  (± 0.2) | 7.8  (± 0.3) | 8.9  (± 0.4) | 7.5  (± 0.4) | 0  (± 0) | 0  (± 0) | 0  (± 0) | 0  (± 0) |  |
| **3** | 37.4  (± 5.6) | 19.3  (± 2.4) | 36.9  (± 1.3) | 16.2  (± 1.3) | 0  (± 0) | 5.3  (± 0.5) | 0  (± 0) | 10.5  (± 0.4) | 15.2  (± 2.7) | 9.2  (± 0.8) | 10.1  (± 0.3) | 7.6  (± 0.5) | 0  (± 0) | 9.2  (± 0.8) | 0  (± 0) | 1.7  (± 0.3) |  |
| **4** | 30.9  (± 2.3) | 28.6  (± 2.2) | 36.0  (± 3.0) | 29.0  (± 4.0) | 0  (± 0) | 0  (± 0) | 0  (± 0) | 0.94  (± 1.9) | 7.5  (± 0.8) | 7.2  (± 0.4) | 8.0  (± 0.5) | 6.8  (± 0.8) | 0  (± 0) | 0  (± 0) | 0  (± 0) | 0  (± 0) |  |
| **5** | 32.5  (± 2.5) | 26.0  (± 1.3) | 32.4  (± 0.3) | 15.1  (± 0.8) | 0  (± 0) | 0  (± 0) | 0  (± 0) | 7.4  (± 0.2) | 7.2  (± 0.7) | 5.9  (± 0.6) | 7.3  (± 0.8) | 1.9  (± 0.2) | 0  (± 0) | 0  (± 0) | 0  (± 0) | 1.6  (± 0.1) |  |

**Online Resource table 5: Ability of tested faecal bacterial isolates to convert glucoraphanin to glucoerucin**

| **Isolate code** | **Bacterial taxa** | **Glucosinolate (mM)** | | **% conversion** |
| --- | --- | --- | --- | --- |
|  |  | *Glucoraphanin* | *Glucoerucin* |  |
| **Control** | n/a | 5.36 | 0 | 0 |
| **1A09** | *Lactobacillus fermentum* | 4.79 | 0 | 0 |
| **1B02** | *Escherichia sp.* | 1.14 | 3.97 | 77.7 |
| **1B03** | *Escherichia sp.* | 0.56 | 4.55 | 89.0 |
| **1B04** | *Escherichia coli* | 0.62 | 4.10 | 86.9 |
| **1C03** | *Enterococcus faecium* | 4.39 | 0 | 0 |
| **1C04** | *Lactobacillus fermentum* | 4.87 | 0 | 0 |
| **1C06** | *Escherichia sp.* | 0.79 | 4.13 | 83.9 |
| **My-0105** | *Enterococcus durans* | 4.75 | 0 | 0 |
| **My-0201** | *Lactobacillus fermentum* | 4.64 | 0 | 0 |
| **My-0302** | *Lactobacillus fermentum* | 4.78 | 0 | 0 |


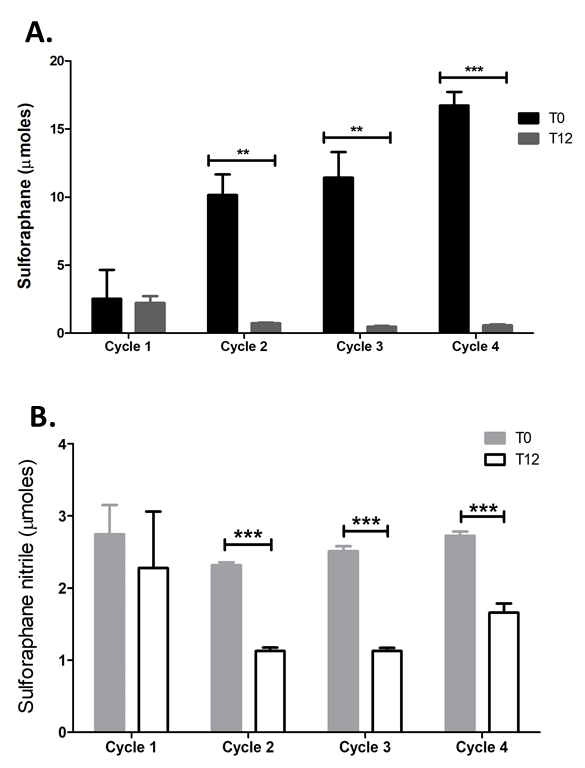


Online Resource figure 1: Levels of sulforaphane (A) and sulforaphane-nitrile (B) in the media when cultured with a human faecal microbiome. Human faecal microbiomes were cultured in a broccoli leachate-containing (BL) media for four 12 hr cycles at 37˚C, under anaerobic conditions.

A. Sulforaphane, and B. Sulforaphane-nitrile in the BL media when cultured with the faecal microbiome of donor 5, which is representative of the observations of all five microbiomes tested. Glucosinolate hydrolysis products were measured using LC-MS/MS. T0; prior to inoculation: T12; 12 hr post-inoculation. Data shown = mean ± SD of four technical replicates. The data was statistically analysed using paired Student’s t-tests (two-tailed) with GraphPad Prism 5.04. ***p*<0.01; ****p*<0.001 zero hr vs 12 hr.


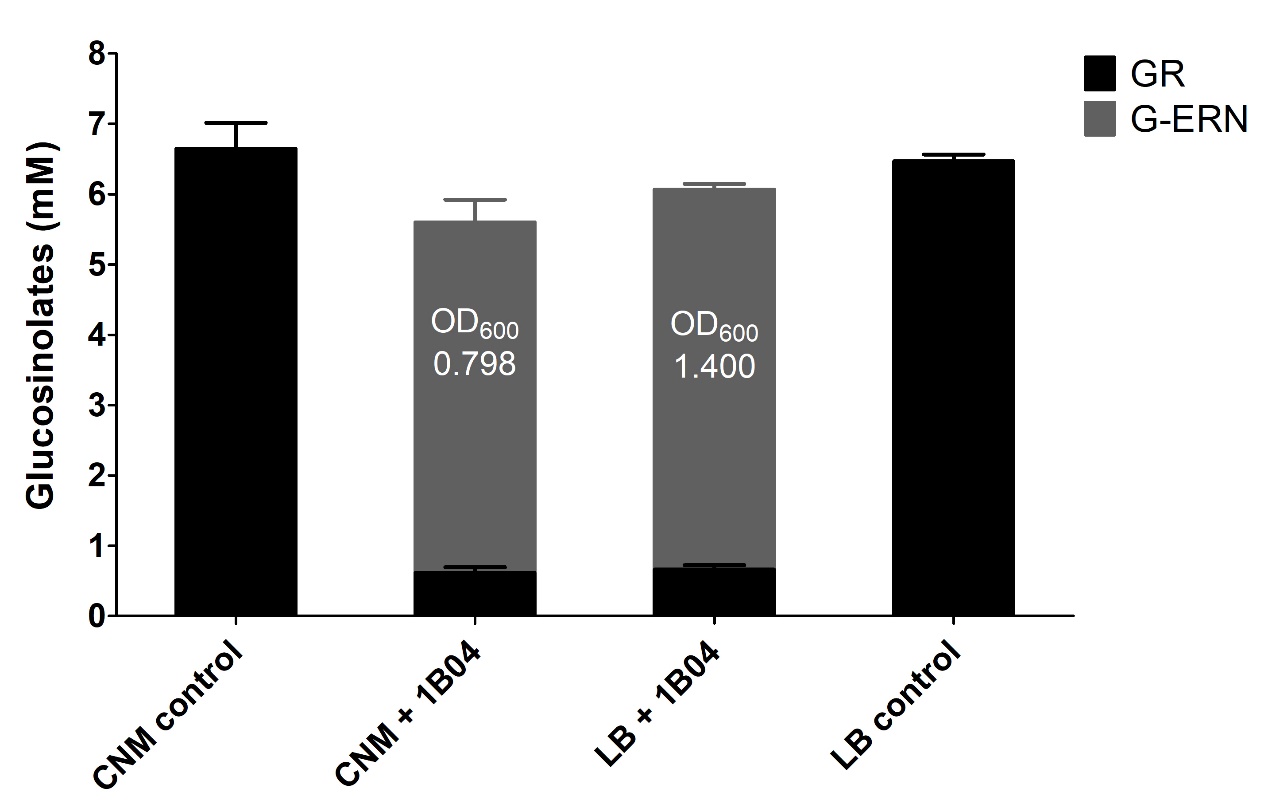


Online Resource figure 2: Comparable levels of glucoraphanin and glucoerucin are observed within both media cultured with *Escherichia coli* 1B04 for 72 hr. *E. coli* 1B04 (5 μl) was cultured in two media (CNM and LB) containing ~6 mM purified glucoraphanin extract (995 μl) for 72 hr at 37˚C, under anaerobic conditions. The control samples consisted of the equivalent media with sterile water (5 μl) added in place of the bacterial inoculum. Glucosinolates were converted to desulphoglucosinolates, measured using HPLC, and analysed with GraphPad Prism 5.04. GR = Glucoraphanin; G-ERN = Glucoerucin; CNM = Chemostat nutrient media; LB = L broth; 1B04 = *Escherichia coli* 1B04. Data shown = mean ± SD of two technical replicates.


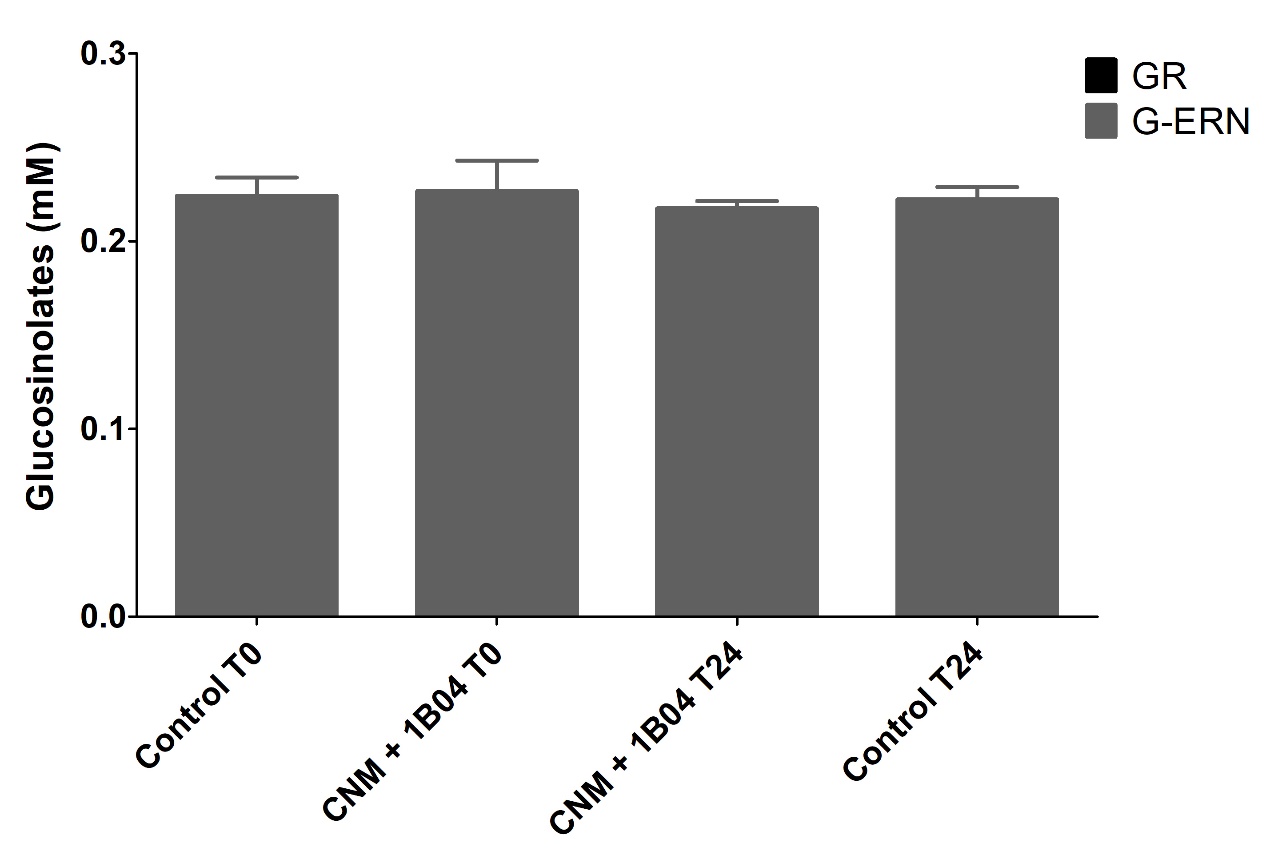


Online Resource figure 3: Glucoerucin is neither oxidised nor hydrolysed by *Escherichia coli* 1B04. *E. coli* 1B04 (5 μl) was cultured in CNM containing ~0.3 mM glucoerucin (995 μl) for 24 hr at 37˚C, under anaerobic conditions. The control samples had sterile water (5 μl) added in place of the bacterial inoculum. Glucosinolates were converted to desulphoglucosinolates, measured using HPLC, and analysed with GraphPad Prism 5.04. GR = Glucoraphanin; G-ERN = Glucoerucin; CNM = Chemostat nutrient media; 1B04 = *Escherichia coli* 1B04; T0 = prior to inoculation; T24 = 24 hr post-inoculation. Data shown = mean ± SD of three technical replicates.
